# The Times–Divide Expression: An Intuitive Approach for Describing Right-Skewed Data in Nursing Practice

**DOI:** 10.1155/jonm/3434734

**Published:** 2025-11-14

**Authors:** Haruna Fukushige, Yoshiaki Inoue, Keisuke Nakashima, Atsue Ishii, Yoko Taniura, Tomoyuki Iwasaki

**Affiliations:** ^1^Graduate School of Health Sciences, Kobe University, Kobe, Hyogo, Japan; ^2^Graduate School of Engineering, The University of Osaka, Osaka, Japan; ^3^Graduate School of Medicine, The University of Osaka, Osaka, Japan; ^4^Division of Nursing, The University of Osaka Hospital, Osaka, Japan

**Keywords:** arithmetic mean and standard deviation, clinical nursing practice, data utilization, data visualization, right-skewed distributions, times–divide expression

## Abstract

In clinical nursing practice, it is crucial to share data overviews simply and intuitively with all team members. Descriptive statistics, typically expressed as the arithmetic mean plus–minus the standard deviation (Mean ± SD), are commonly used for this purpose. However, this approach is inadequate for describing asymmetric, right-skewed distributions, commonly encountered in nursing. This study introduces an alternative—the times–divide expression (GMean ^×^/GSD)—based on the geometric mean and geometric standard deviation. We present a data-driven study that examines the applicability of this expression using nursing demand data collected over four and a half years from a nurse call system. The results indicate that the times–divide expression outperforms conventional methods in representing distributional properties and providing suitable representative values and usable percentile range indicators. Applying the times–divide expression to the actual distribution of data is expected to improve nurses' clinical decision-making skills through data-driven insights, ultimately enhancing the quality of care.

## 1. Introduction

In clinical nursing practice, the use of data has become increasingly important for improving the quality of patient care. The data collected in hospitals are both large and longitudinal, offering the potential to provide a richer understanding of patients and to tailor personalized intervention strategies to each individual [1, 2]. Knowledge is crucial for enhancing the quality of clinical judgments, which nurses must make multiple times a day and which significantly influence patient outcomes [3–5]. For instance, knowledge has been reported to outweigh experience in improving the accuracy of triage decisions in emergency departments [6]. These findings suggest that by acquiring more knowledge not only from experience but also through data, nurses can make more accurate clinical judgments, tailor personalized interventions, and ultimately improve patient outcomes.

In other words, when data are used in clinical nursing practice, it is important not only to present detailed information about complex phenomena using advanced mathematical and statistical techniques, but also to provide an overview of the data in a simple and intuitive way accessible to all members of the clinical nursing team.

Additionally, nurses need to understand the distribution of data, as it reveals both typical trends and variations in patient conditions. This understanding allows them to identify what constitutes a typical pattern and how individual patients conform to or deviates from that pattern. By recognizing these differences, nurses can distinguish between expected clinical presentations and those that require special attention, thereby supporting the delivery of personalized care. For convenience, we define the set of target phenomena that nurses seek to represent using data as “nursing phenomena.” Overall, effective use of data in clinical practice requires the simple and intuitive representation of the distribution of nursing phenomena.

**Figure 1:**
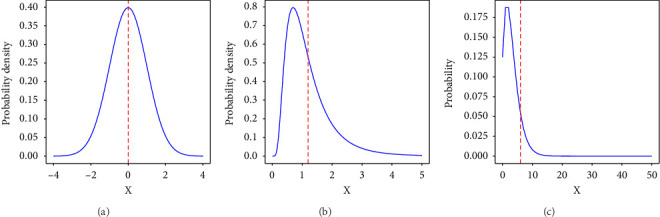
Various types of distributions. The blue line indicates the data, and the red dashed line is the mean. (a) Normal distribution. (b) Log-normal distribution. (c) Negative binomial distribution.

The arithmetic mean (Mean) and standard deviation (SD), commonly expressed as Mean ± SD, are often used to describe data simply and intuitively to capture both distribution and variance, particularly in nursing research. However, this approach is often unsuitable for data exhibiting an asymmetric, right-skewed distribution [7, 8]. The Mean and SD assume a symmetric distribution, similar to the normal distribution. The symmetry in the distribution indicates that the frequency of data samples is equal in both smaller and larger directions from the representative value, typically indicated by the mean (Figure 1(a)). For example, in a symmetric distribution, the number of patients requiring less nursing care than average would equal the number of patients requiring more care.

Many clinical nurses routinely feel that most patients require only minimal nursing care, whereas a smaller group requires extensive care [9–11]. Such impressions indicate that the underlying data distributions may have longer right tails, suggesting conformance to asymmetric right-skewed distributions, such as the log-normal (Figure 1(b)) and negative binomial (Figure 1(c)) distributions. Right-skewed distributions are frequently observed in nursing-related data, such as the length of patient stay [12], time required for patient care [13], fall risk scores [14], and pressure on patients' lumbar spines [15]. We note that the right-skewed distributions are also often referred to as long-tailed distributions, although the strict mathematical definition of long-tailedness is based on the asymptotic decay rate of tails. Therefore, the routine use of Mean ± SD can be problematic in describing right-skewed data in nursing practice.

Two specific problems arise when using the expression Mean ± SD in describing data with right-skewed distributions. First, the Mean tends to take a value larger than the representative value of the population and deviate from the actual situation. The dashed red line in Figure 1 represents the mean value. For the log-normal (Figure 1(b)) and negative binomial (Figure 1(c)) distributions, we observe that the mean takes a value larger than the representative value; the right-skewed distribution is heavily influenced by outliers, causing the mean to deviate upward from the representative value. Second, the range indicated by Mean ± SD is often wide. Since the SD is highly influenced by outliers, the resulting range may include nearly all sample values, making it almost meaningless—or worse, misleading interpretations. A more significant issue arises when the lower half of the range [Mean − SD, Mean] includes negative values, which actual samples cannot take. This issue is inherent to the Mean ± SD expression, which assumes a symmetric distribution. Consequently, the commonly used Mean ± SD expression may fail to accurately represent the characteristics of data with right-skewed distributions. Therefore, it limits nurses' ability to understand the true statistical properties of the data and hinders the effective use of data in clinical practice.

To address these issues, this study proposes using the times–divide expression instead of the conventional Mean ± SD for right-skewed distributions. The times–divide expression, denoted as geometric mean (GMean) ^×^/geometric standard deviation (GSD) [7, 8], is a well-established method for describing probability distributions in terms of the GMean and GSD. This method has garnered attention in various scientific fields [16–24] as an effective way to describe log-normally distributed data, which is one of the typical types of right-skewed distributions. Similar to the conventional Mean ± SD, the times–divide expression GMean ^×^/GSD provides a simple and intuitive method for indicating the representative value and a unit range of dispersion using only two parameters. While GMean ^×^/GSD is likely more suitable for representing right-skewed data in nursing practice, it has so far only been validated for log-normal distributions—a specific type of right-skewed distribution with a strict assumption of log-normality. However, nursing data do not always follow a log-normal distribution, and thus, the direct application of this method to other types of right-skewed data remains uncertain.

Therefore, this study examines the usefulness of the times–divide expression using real-world nursing data that are right-skewed but do not follow a log-normal distribution. To this end, this study presents real-world data using both the conventional Mean ± SD and the times–divide expression, and it compares them for suitability. It is anticipated that this study's findings will assist clinical nurses in making better clinical decisions through more effective use of data.

The purpose of this study is to demonstrate that the times–divide expression is more suitable than the Mean ± SD expression for representing nursing phenomena characterized by right-skewed, but not necessarily log-normal, distributions. By proposing a more suitable method for representing such data, this study aims to support nursing managers in effectively utilizing the data they already have.

## 2. Methods

### 2.1. Times–Divide Expression

The times–divide expression, originally proposed by Limpert et al., is known as a key tool for representing the log-normal distribution, a typical right-skewed non-normal distribution [7, 8]. Although the times–divide expression has not received much attention in medical research, its application is gaining prominence in various fields of science [16–24]. In contrast to the traditional expression of Mean ± SD, this approach uses the GMean and GSD, and it describes the data in the form of GMean ^×^/GSD, where the sign ^×^/is read as times or divided. According to the method proposed by Limpert et al. [7, 8], GMean and GSD are calculated by first taking the logarithm of the data and then computing the mean and SD of the log-transformed values. The methods for calculating the mean and SD remain essentially the same as those used in standard arithmetic calculations. The range of dispersion is then represented by the lower and upper limits GMean/GSD and GMean × GSD. Therefore, this distinction allows the variance to be expressed in a multiplicative manner (times or divided, ×/), as opposed to the additive (plus or minus, ±) representation observed in standard methods. The times–divide expression is suitable for describing right-skewed distributions because its multiplicative nature enables us to represent a range, which is asymmetric around the representative value GMean.

The use of GMean and GSD in the times–divide expression follows the approach proposed by Limpert et al. [7, 8]. For data *X* = {*X*_1_, *X*_2_,…, *X*_*N*_}(*X*_*i*_ > 0,  *i* = 1, 2,…, *n*), the GMean *m* and GSD *s* are defined as the exponentials of the mean and SD of the log-transformed data {log(*X*_1_), log(*X*_2_),…, log(*X*_*N*_)}:

(1) $$\begin{array}{c} m=\exp\left(\frac{1}{N}\overset{N}{\underset{k=1}{\sum }}\log\;X_{k}\right),\\ s=\exp\left(\sqrt{\frac{1}{N-1}\overset{N}{\underset{k=1}{\sum }}{\left(\log X_{k}-\log m\right)}^{2}}\right). \end{array}$$

For comparison, the ordinary Mean *μ* and SD *σ* are given by

(2) $$\begin{array}{c} \mu =E\left[X\right]=\frac{1}{N}\overset{N}{\underset{k=1}{\sum }}X_{k},\\ \sigma =\text{SD}\left[X\right]=\sqrt{\frac{1}{N-1}\overset{N}{\underset{k=1}{\sum }}{\left(X_{k}-\mu \right)}^{2}}. \end{array}$$

Although *s* is not strictly the GMean of the squared deviations, we follow conventional terminology and refer to it as GSD.

In this framework, the GMean ^×^/GSD corresponds to the range indicated by the Mean ± SD of the log-transformed data. The interval corresponding to one SD in log-space, namely, *μ* ± *σ*, transforms back to [*m*/*s*, *m* × *s*]. Likewise, the interval corresponding to two SDs in log-space, *μ* ± 2*σ*, transforms back to [*m*/*s*^2^, *m* × *s*^2^]. For clarity, we call *μ* ± *σ* the plus-minus expression, and *m*^×^/*s* the times–divide expression.

Since the definitions of GMean and GSD rely on a logarithmic transformation, the framework requires that all observed values *X*_*k*_ are strictly positive. In cases where zero or negative values are present, appropriate handling—such as modeling the proportion of zeros separately or applying non-negative transformations to negative values—should be considered based on the analytical objective. A detailed treatment of such cases, however, lies beyond the scope of this study and is left for future work.

### 2.2. Data

#### 2.2.1. Nursing Phenomenon Data

The number of times patients called the nurse was used as a representation of the complex and chaotic aspects of nursing care. Patients can call nurses through two methods other than direct communication: intentional use of the nurse call button found on the patient's bed and automatic alerts triggered by devices that send notifications to nurses without the patient's intention, which notifies nurses of events such as the patient leaving the bed or experiencing an arrhythmia. The former mode of communication is available to all patients who can push the button, although many use it only a few times a day. The latter is only available to patients who have a device that generates an alarm and is often used repeatedly throughout the day, as alarms for events such as getting out of bed or having an arrhythmia can occur several times. These two methods have different causes and mechanisms, and each has unique characteristics. Specifically, the number of times a patient called the nurse can be validated using two different types of data: nurse calls and sensor alerts.

Both the nurse call and the sensor alert data used in this study are recorded in the nurse call log, which serves as a comprehensive historical record of usage. Due to their inherent characteristics, these logs are particularly well-suited to analyzing large datasets. This is mainly because the data can be systematically collected over long periods without omissions, ensuring a complete and reliable dataset for analysis. Therefore, the present study analyses the number of times these two types of patients called for a nurse.

#### 2.2.2. Data Source

The target institutions were university hospitals. The target data were collected from April 1, 2016, to September 22, 2020. A total of 23 wards were examined, except for perinatal wards, which had different nurse call systems. The total number of eligible nurse call logs was 5, 116, 598.

#### 2.2.3. Data Processing

The frequency of use per patient per day for each data type was calculated using the following two data processing steps: First, the nurse call logs were categorized by call type into nurse call data and sensor alert data. Second, the number of calls generated per patient per day was counted within each data type. The same patient was counted as a different person on the following day. The final data count was 650, 147 calls for the nurse call dataset and 49, 042 calls for the sensor alert dataset.

**Figure 2:**
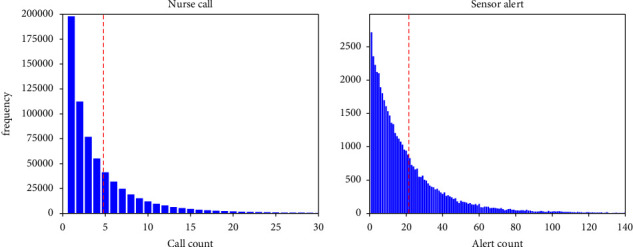
Distribution of target data. The blue line indicates the data, and the red dashed line is the mean.

**Figure 3:**
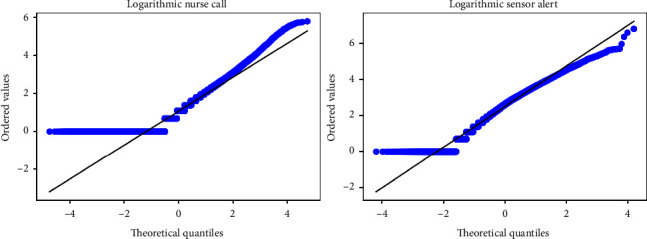
QQ plot of logarithmic target. The blue line indicates the data, and the black line is the reference line.

Finally, the shape of the distribution was confirmed. Upon visually inspecting the shape of the distribution, it was noted that each dataset exhibited a right-skewed distribution (Figure 2). The fact that the distributions were not log-normal was confirmed using quantile–quantile (QQ) plots after applying a logarithmic transformation to the data. The results showed that neither dataset followed a log-normal distribution (Figure 3).

### 2.3. Verification Method

As previously mentioned, two major problems exist with the ordinary plus-minus expression. The first problem is that the mean is significantly affected by the length of the tail of the distribution, resulting in a value larger than the representative value of the distribution. To evaluate this aspect, the validity as a representative value was investigated using the median of the data as the criterion. More specifically, we examined the distance of the representative values (Mean *μ* and GMean *m*) from the median to quantify their adequacy. The second problem we focus on is the validity of the dispersion range indicated by the expressions. A range that is too wide can contain almost all values of samples or include nonexistent ranges, such as negative values, making it almost useless for clinical purposes. In contrast, if the range is too narrow, it fails to express the overall variations between patients, making it also clinically useless. In summary, a range that is neither excessively broad nor unduly narrow is deemed appropriate.

To evaluate this aspect, we investigate the adequacy of the range as follows. In the normal distribution, the ordinary use of the plus–minus expression assumes (if implicitly) that the range between the percentiles one unit (two units, respectively) away from the center covers 68% (95%, respectively) of the whole distribution, which is the representative property of the normal distribution. In this study, therefore, the range from the top 16th percentile point to the top 84th percentile point (68% of the total) was defined as one unit of intuitive coverage, and the range from the top 2.5th percentile point to the 97.5th percentile point (95% of the total) as two units of intuitive coverage. We then evaluate the similarity between this intuitive coverage and the ranges indicated by plus–minus or times–divide expressions.

To be more specific, the similarity was verified utilizing the Jaccard index. The Jaccard index is a widely used method to verify the similarity of discrete sets and is also used in the medical field as a method to verify similarity [25, 26]. The Jaccard index was calculated for the two intervals A (a, b) and B (c, d) using the following equation:

(3) $$\begin{array}{c} \text{Jaccard index}=\frac{\left|A\cap B\right|}{\left|A\cup B\right|}\times 100\\ =\frac{\min\left(b,d\right)-\max\left(a,c\right)}{\max\left(b,d\right)-\min\left(a,c\right)}\times 100. \end{array}$$

**Figure 4:**
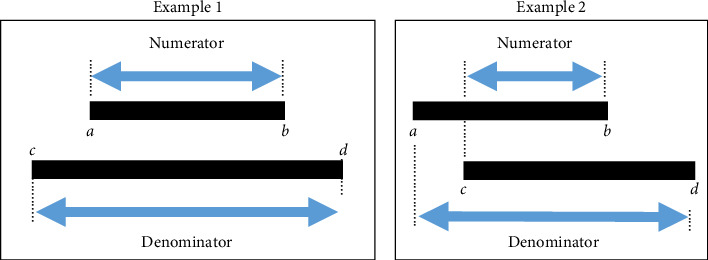
Example of Jaccard index calculation.

The Jaccard index is defined as the size of the intersection of the two sets divided by the size of the union of the two sets (Figure 4). In Figure 4, the denominator is larger, and the numerator is smaller in Example 2 than in Example 1. Hence, the similarity is greater in Example 2 than in Example 1. In this study, the similarity was verified by calculating the Jaccard index between the interval indicated by the normal distribution and each expression. Thus, in this study, we compare the suitability of the times–divide expression and the plus–minus expression, using two key indicators—the validity of a representative value and the similarity of the adequacy of the dispersion range.

## 3. Results

All hospital wards were included in this study. However, in the sensor alert dataset, accurate values were difficult to obtain for four wards because fewer than 100 patients, and eligibility was restricted to patients attached to a monitoring system that had a sensor. Therefore, the following four wards were excluded from the sensor alert results: Emergency A (*n* = 74), Emergency B (*n* = 75), Pediatrics (*n* = 8), and Pediatrics surgery (*n* = 7).

### 3.1. Overview of the Results for a Representative Value and a Range

**Figure 5:**
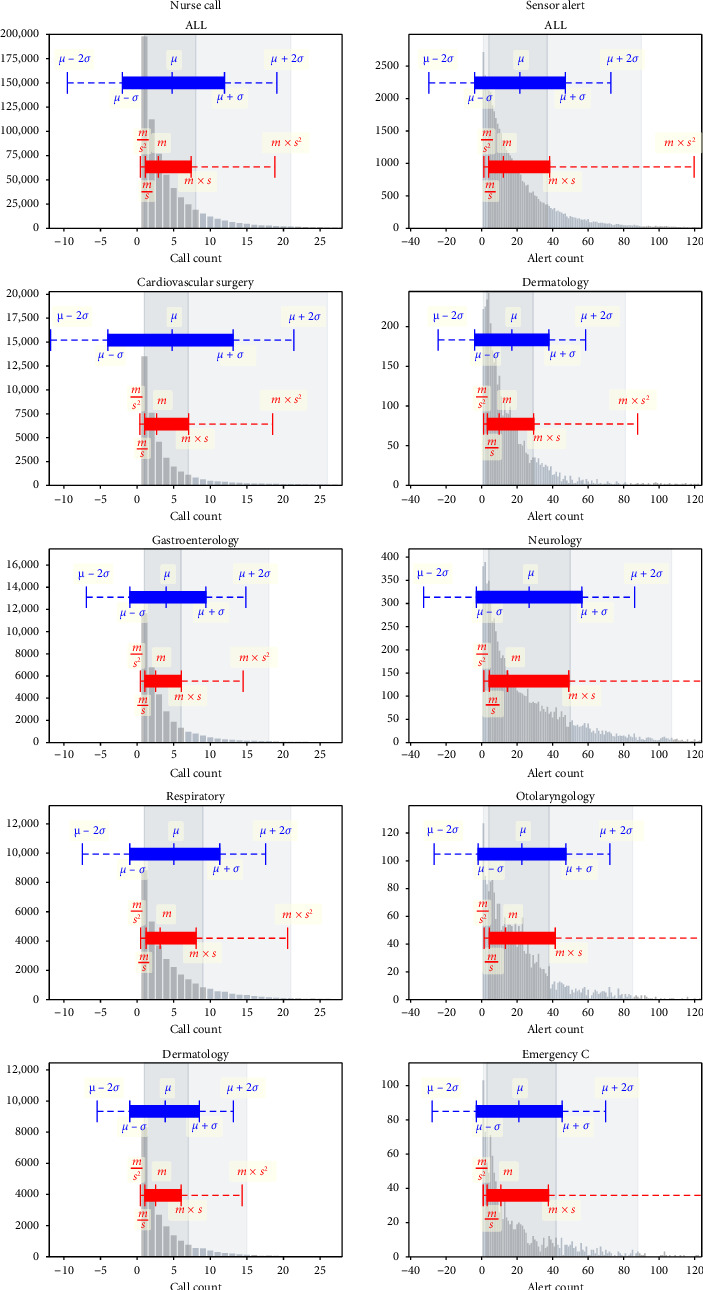
Overview of the results for a representative value and a range. The blue parts show the mean and standard deviation (*μ* ± *σ*) results, and the red parts indicate the times–divide (*m*^×^/*s*) results. The gray background shows intuitive coverage. Owing to the limitations of paper width, the data shown for each ward are representative of the top two wards that were highly similar in each dataset and the bottom two wards that were less similar. The rest of the data are presented as Supporting Information.

To obtain an overall picture, the histogram of *X* and the ranges indicated by the times–divide expression and by the plus–minus expression are shown for the nurse call data and the sensor alert data (Figure 5). All ward data were analyzed. However, due to space limitations, the data shown for each ward are representative of the following four wards in each dataset: the top two wards with the highest similarity and the bottom two wards with the lowest similarity for the one-unit result. Additional data are presented in the Supporting Information (available here).

In the plus–minus expressions, two of the aforementioned problems—a larger mean than the representative value and a too-wide variance range—were observed. For the representative values of *m* in the times–divide expression and *μ* in the plus–minus expressions, *μ* was greater than *m* for all wards in both datasets. For the range of values shown, the plus–minus expressions gave negative values for all wards in both datasets. The times–divide expression does not yield negative values, as it is derived from a logarithmic transformation.

### 3.2. Validity as a Representative Value

Table 1 shows the deviations of *m* in the times–divide expression and *μ* in the plus–minus expression from the median. Across both datasets, the absolute deviation from the median was smaller for *m* than for *μ*. In the combined results, the absolute deviation of *m* was 1/18 that of *μ* in the nurse call dataset and 1/4 that of *μ* in the sensor alert dataset. In the nurse call dataset, the absolute deviation of *m* from the median remained below one in all cases.

### 3.3. Similarity

Table 2 presents the similarity of all data and data for each ward. Except for the two units of data from the ophthalmology ward in the sensor alert dataset, the similarity for the times–divide expression was higher than for the plus–minus expressions for all other results.

In the nurse call dataset, the similarity between the range shown by the times–divide expression and intuitive coverage was a maximum of 99.1 and a minimum of 82.7 for the one-unit results and a maximum of 98.5 and a minimum of 66.6 for the two-unit results. The similarity between the range shown by the plus–minus expressions and intuitive coverage was a maximum of 69.3 and a minimum of 22.4 for the one-unit results and a maximum of 66.1 and a minimum of 37.2 for the two-unit results.

In the sensor alert dataset, the similarity between the range shown by the times–divide expression and intuitive coverage was a maximum of 98.3 and a minimum of 88.5 for the one-unit results and a maximum of 94.2 and a minimum of 61.4 for the two-unit results. The similarity between the range shown by the plus–minus expressions and intuitive coverage was a maximum of 81.9 and a minimum of 51.7 for the one-unit results and a maximum of 66.2 and a minimum of 55.3 for the two-unit results.

## 4. Discussion

Right-skewed distributions, which are not always log-normal, are frequently observed in nursing-related data. This study aims to demonstrate that the times–divide expression is more suitable than the plus–minus expression for representing such distributions. Specifically, the times–divide expression addresses two key limitations of the plus–minus expression: the tendency to produce a mean greater than the representative value and an excessively wide range of variation. In our analyses, the times–divide expression consistently offered a more appropriate and intuitive representation of such distributions.

For the first problem, the validity of each expression as a representative value was examined based on its absolute deviation from the median, a common representative value in normal distributions. We found that the absolute deviation between each representative value and the median was smaller for the times–divide expression than for the plus–minus expression. In addition, the absolute deviation in the times–divide expression in the nurse call dataset was less than one for all results, indicating that it closely approximates the median, especially given that nurse calls are discrete values. These results suggest that the times–divide expression provides a more appropriate representative value than the plus–minus expression.

Moreover, the representative value of the times–divide expression was almost equal to the median for the nurse call dataset, whereas it was smaller than the median for the sensor alert dataset. This result shows that the representative values of the times–divide expression were less likely to be unduly influenced by some outliers. The two datasets used in this study had different characteristics. The nurse call dataset showed a relatively convergent distribution, where most patients remained at small values of 25 or less, even though all patients were covered. Conversely, the sensor alert dataset was characterized by a large number of uses per patient and a high variance, even though the number of patients was small because only patients with a sensor setup were included. In other words, the sensor alert dataset was more likely to be affected by patients who triggered more alerts than the nurse call dataset. The representative value of the times–divide expression being smaller than the median in the sensor alert dataset, which is susceptible to some outlier patients, indicates that the times–divide expression was less affected by outlier patients than the median. The representative values must represent the characteristics of most patients without being unduly influenced by a few extreme cases. The datasets in this study had a minimum value of one occurrence as the most common value. This implies that smaller values are closer to the representative value. These considerations suggest that the times–divide expression can provide highly valid representative values even for distributions with large variability and susceptibility to some outliers.

For the second problem, the adequacy of the range shown was evaluated based on how closely it matched intuitive coverage. The similarity was much higher for the times–divide expression than for the plus–minus expression across all datasets, except for two units in the data from the ophthalmology ward. This difference is also apparent in the visualization of the overview (Figure 5). The low similarity in the data from the ophthalmology ward is due to the large value of *m*^×^/*s*^2^, as shown in Figure 5. The low values on the *y*-axis of Figure 5 show that this ward had a relatively small dataset. Despite some participants having over 80 alerts per day, the overall distribution had a more moderate shape than the distributions in the other wards. This indicates that the distribution of the data from the ophthalmology ward was highly variable. It is important to note that the range shown reflects variability. While it is crucial for representative values to minimize the influence of some patients and focus on the majority trend, it is equally important for the range to capture variability by reflecting the contributions of all patients, including those with extreme values. The range derived from the times–divide expression better captured the variability of the distribution than the intuitive coverage did.

The reason why the quantile range can be effectively represented using the times–divide expression is that, when the data are log-transformed, it tends to become nearly symmetric around the mean. On the other hand, when examining the histogram of the log-transformed data, it can be observed that the right tail is longer than the left tail, indicating that the distribution is not perfectly symmetric. Nevertheless, in practice, it is important to note that the log transformation significantly stretches values on the left tail of the distribution. Consequently, the detailed shape of the left tail in the log-transformed histogram has a diminished impact on the quantiles of the original scale. In other words, if there were two datasets under the log transformation where the right tail is equal and only the left tail differs, they would have nearly identical quantiles in their original (non-log-transformed) scale. Therefore, in the descriptive statistics of right-skewed data, even if the data do not follow a log-normal distribution, the times–divide expression is likely preferable to the plus–minus expression.

What is important is to represent data using a method that aligns with its underlying distribution. While the conventional plus–minus expression is appropriate for normally distributed data, the findings of this study indicate that the times–divide expression is more suitable for right-skewed distributions, even when they are not strictly log-normal. Therefore, the times–divide expression should be regarded as an equally important option as the plus-minus expression in descriptive statistics, and the choice between them should be based on the actual shape of the data distribution. We believe that this approach will enable more effective use of data in nursing practice. Furthermore, we believe that a proper understanding of data distribution and the use of appropriate representation methods will be crucial in more advanced applications of data, such as factor analysis and predictive modeling, which are likely to become increasingly important in the future.

In summary, the results showed that the times–divide expression was more suitable than the plus–minus expression from all perspectives, even for right-skewed distributions that did not exhibit log-normal characteristics. Furthermore, it was shown that the values indicated by the times–divide expression have characteristics such that the representative value is less influenced by some outlier patients, while the range reflects the attributes of some outlier patients. This is particularly important, as most nursing phenomena are likely to exhibit such skewed distributions.

## 5. Implications for Nursing Management

The importance of data utilization in nursing management is well-recognized [27–29]. However, in practice, implementing data-driven approaches can be challenging, and many nursing managers may struggle to effectively use the data they have. One possible reason is that, although the Mean ± SD is a useful method in many contexts, it is often unsuitable for the types of data commonly encountered in nursing, limiting the ability to extract meaningful insights. The core issue is the mismatch between familiar methods and the specific characteristics of the data. This study proposes a method tailored for right-skewed distributions, which are frequently observed in nursing data, and aims to address this issue. Essentially, this method can be readily adopted by all team members, facilitating the sharing of information across the nursing team. Therefore, this study proposes a method that succinctly and intuitively represents right-skewed distributions.

Demonstrating nursing phenomena in a way that all nurses can intuitively understand may significantly improve the quality of nursing care. For example, the sensor alert dataset, as indicated by the times–divide expression, showed that the representative value was 12.2 times/day. The one-unit range was from 3.9 to 38.3 times/day, and the two-unit range was from 1.2 to 119.7 times/day. From these results, nurses can infer that the typical patient experienced sensor calls 12.2 times/day, approximately 70% of patients experienced between 3.9 and 38.3 sensor calls per day, and almost the entire patient population experienced sensor calls between 1.2 and 119.7 times/day. As previously mentioned, knowledge is crucial for improving the appropriateness of clinical decisions. For example, deciding who should be equipped with alarm sensors and limiting their use to the minimum necessary patients is essential for controlling the number of calls across the ward. A clear and intuitive understanding of the daily call distribution, supported by times–divide expressions, can enhance the accuracy of nurses' decisions regarding sensor placement. By integrating data-driven insights with nurses' clinical experience, times–divide expressions can significantly improve the quality of clinical decision-making.

Furthermore, we believe that this effect may be more pronounced for novices than for experts because experts are better at reading meaningful patterns from information and organizing knowledge than novices [30]. Specifically, compared to expert nurses, novices have less information about nursing phenomena, such as patterns and situational awareness, even when they have experienced the same situation. Data may help novices improve their judgment by acquiring information not obtained through experience. We believe that the data support these informational differences and contribute to the improvement of novice nurses' skills. The education of novice nurses requires a significant amount of time and effort, and the burden on new nurses during the learning process is also substantial. We believe that this method can contribute to alleviating these burdens through the effective use of data.

The development of digital technology, such as Internet of Things (IoT) devices, has made a variety of data available. Nursing must leverage these data to advance the discipline. Future nursing managers need strong information competency, and nursing managers and researchers must develop a deeper understanding of actuarial and statistical knowledge [31]. Concurrently, as nursing is a team activity, if all nurses can gain more knowledge and a greater sense of what it means to be a nurse, this may improve the nursing skills of the team and enable the provision of higher quality nursing care to more patients. This study proposes a simple and intuitive way for all nurses to imagine data as one way to utilize data. By employing this approach, nursing practice can be better supported with the data now available.

## 6. Limitations

This study has two major limitations. First, it employs two datasets from only a single institution. To address this limitation, we presented ward-specific data in Tables 1 and 2, and included additional graphs in the Supporting Information that are not shown in Figure 5. This approach allows examination of results across datasets with varying call usage patterns and frequencies, accounting for differences in distribution shapes and sample sizes. In addition, the Supporting Information includes further analyses using random sampling with varied sample sizes. These analyses demonstrate that the times/divide representation yields more consistent and robust results than the plus–minus representation, particularly in smaller datasets. Nonetheless, further investigation is needed to determine whether these findings can be replicated across other datasets.

Second, the times–divide expression can only be used for data with more than a one-time because it uses logarithms. The validity of the times–divide expression for data significantly influenced by zero-time data requires further research. Given these limitations, future research should explore the application of the times–divide expression to different types of data and validate its effectiveness.

## 7. Conclusions

Comparative validation of the times–divide and mean ± SD expressions using the nurse call and sensor alert datasets strongly suggests that the times–divide expression is more suitable for representing nursing phenomena in all validated items. Furthermore, it was shown that the representative values given by the times–divide expression are less influenced by some outlier patients, while the range reflects the characteristics of some outlier patients. We believe that this descriptive method can contribute to improving the quality of nursing care by presenting the characteristics of nursing phenomena in a simple and intuitive way for all nurses to support their clinical judgment.

## Figures and Tables

**Table 1:** Deviations of the representative value of the times–divide expression and the plus–minus expressions from the median of all data and data by ward in each dataset.

Hospital ward (nurse call (*n*)/sensor alert (*n*))	Nurse call			Sensor alert		
	Median	*m* ^×^/*s* [*m* − med]	*μ* ± *σ* [*μ* − med]	Median	*m* ^×^/*s* [*m* − med]	*μ* ± *σ* [*μ* − med]
ALL (650, 147/49, 042)	3.0	2.9^**×**^/2.5 [−0.1]	4.8 ± 7.2 [+1.8]	14.0	12.2^**×**^/3.1 [−1.8]	21.5 ± 25.6 [+7.5]

Emergency C (7779/1208)	5.0	4.6^**×**^/2.8 [−0.4]	7.9 ± 10.5 [+2.9]	12.0	10.8^**×**^/3.5 [−1.2]	20.9 ± 24.4 [+8.9]

Neuropsychiatry (15, 330/2160)	3.0	3.8^**×**^/3.3 [+0.8]	8.4 ± 15.9 [+5.4]	11.0	10.0^**×**^/2.7 [−1.0]	15.5 ± 20.1 [+4.5]

Nephrology (19, 982/2184)	2.0	2.4^**×**^/2.6 [+0.4]	4.1 ± 6.1 [+2.1]	16.0	13.9^**×**^/3.0 [−2.1]	22.9 ± 25.4 [+6.9]

Obstetrics and gynecology (27, 216/709)	2.0	2.4^**×**^/2.3 [+0.4]	3.6 ± 4.3 [+1.6]	8.0	7.1^**×**^/2.8 [−0.9]	11.5 ± 12.8 [+3.5]

Respiratory (32, 325/2901)	3.0	3.2^**×**^/2.6 [+0.2]	5.0 ± 6.3 [+2.0]	11.0	9.8^**×**^/2.8 [−1.2]	15.7 ± 15.7 [+4.7]

Neurology (34, 677/7933)	4.0	3.7^×^/2.8 [−0.3]	6.4 ± 9.6 [+2.4]	17.0	14.6^**×**^/3.4 [−2.4]	26.8 ± 29.7 [+9.8]

Cardiovascular medicine (38, 699/1329)	2.0	2.6^**×**^/2.7 [+0.6]	4.8 ± 8.3 [+2.8]	15.0	13.5^**×**^/3.0 [−1.5]	22.6 ± 24.4 [+7.6]

Hematology (38, 610/671)	3.0	3.0^**×**^/2.3 [±0.0]	4.4 ± 5.7 [+1.4]	16.0	15.1^**×**^/3.5 [−0.9]	29.7 ± 37.8 [+13.7]

Gastroenterology (34, 025/3068)	2.0	2.5^**×**^/2.4 [+0.5]	4.0 ± 5.4 [+2.0]	14.0	12.3^**×**^/2.9 [−1.7]	19.8 ± 19.0 [+5.8]

Diabetes medicine (23, 960/1842)	3.0	3.0^**×**^/2.7 [±0.0]	5.3 ± 7.7 [+2.3]	21.0	18.7^**×**^/3.1 [−2.3]	31.4 ± 33.6 [+10.4]

Otolaryngology (33, 967/2248)	3.0	3.4^**×**^/2.4 [+0.4]	5.0 ± 6.1 [+2.0]	16.0	13.4^**×**^/3.1 [−2.6]	22.7 ± 24.7 [+6.7]

Orthopedic surgery (34, 150/626)	2.0	2.7^**×**^/2.4 [+0.7]	4.1 ± 4.5 [+2.1]	15.0	14.2^**×**^/3.0 [−0.8]	24.2 ± 27.4 [+9.2]

Ophthalmology (18, 544/1046)	1.0	1.6^**×**^/1.9 [+0.6]	2.2 ± 4.7 [+1.2]	14.0	11.5^**×**^/3.3 [−2.5]	20.9 ± 30.7 [+6.9]

Dermatology (23, 293/3748)	2.0	2.5^**×**^/2.4 [+0.5]	3.8 ± 4.7 [+1.8]	10.0	9.8^**×**^/3.0 [−0.2]	17.0 ± 20.8 [+7.0]

Cardiovascular surgery (39, 116/1838)	2.0	2.7^**×**^/2.6 [+0.7]	4.8 ± 8.3 [+2.8]	15.0	13.2^**×**^/3.3 [−1.8]	24.8 ± 33.3 [+9.8]

Gastrointestinal Surgery A (42, 624/3229)	3.0	2.9^**×**^/2.4 [−0.1]	4.4 ± 5.5 [+1.4]	11.0	10.0^**×**^/3.0 [−1.0]	17.0 ± 19.7 [+6.0]

Gastrointestinal Surgery B (44, 045/1531)	3.0	3.1^**×**^/2.5 [+0.1]	4.9 ± 7.1 [+1.9]	13.0	10.5^**×**^/3.1 [−2.5]	17.9 ± 19.0 [+4.9]

Urology (29, 527/2794)	2.0	2.4^**×**^/2.3 [+0.4]	3.6 ± 5.0 [+1.6]	13.0	11.6^**×**^/3.2 [−1.4]	20.4 ± 23.8 [+7.4]

Neurosurgery (29, 192/7813)	3.0	3.3^**×**^/2.6 [+0.3]	5.5 ± 7.5 [+2.5]	14.0	13.0^**×**^/3.0 [−1.0]	21.9 ± 24.6 [+7.9]

Emergency A (6018/74)	5.0	5.0^**×**^/2.9 [±0.0]	9.0 ± 12.2 [+4.0]	—	—	—

Emergency B (8844/75)	4.5	4.5^**×**^/3.0 [±0.0]	8.3 ± 12.8 [+3.8]	—	—	—

Pediatrics (43, 426/8)	3.0	3.3^**×**^/2.3 [+0.3]	4.8 ± 5.4 [+1.8]	—	—	—

Pediatrics surgery (24, 751/7)	3.0	2.9^**×**^/2.3 [−0.1]	4.3 ± 4.6 [+1.3]	—	—	—

*Note:* For the sensor alert dataset, for wards with < 100 data points, calculations of the correct similarity could not be performed, and they were therefore excluded from consideration. Numbers in bold indicate smaller differences from the median. *n*: number of data. *m*^×^/*s*: the times–divide expression. *μ* ± *σ*: the plus–minus expressions.

**Table 2:** Similarity of all data and data for each ward.

		Nurse call			Sensor alert		
Hospital ward	Unit	Intuitive coverage	Similarity		Intuitive coverage	Similarity	
		[16^th^, 84^th^] [2.5^th^, 97.5^th^]	*m* ^×^/*s* [*m*/*s*, *m* × *s*] [*m*/*s*^2^, *m* × *s*^2^]	*μ* ± *σ* [*μ* − *σ*, *μ* + *σ*] [*μ* − 2*σ*, *μ* + 2*σ*]	[16^th^, 84^th^] [2.5^th^, 97.5^th^]	*m* ^×^/*s* [*m*/*s*, *m* × *s*] [*m*/*s*^2^, *m* × *s*^2^]	*μ* ± *σ* [*μ* − *σ*, *μ* + *σ*] [*μ* − 2*σ*, *μ* + 2*σ*]
ALL	1-unit	[1.0, 8.0]	89.3 [1.1, 7.4]	50.2 [−2.0, 11.9]	[4.0, 37.0]	96.1 [3.9, 38.3]	64.5 [−4.0, 47.1]
	2-unit	[1.0, 21.0]	86.7 [0.4, 18.8]	59.3 [−9.5, 19.1]	[1.0, 90.0]	74.8 [1.2, 119.7]	59.9 [−29.8, 72.8]

Emergency C	1-unit	[1.0, 13.0]	94.8 [1.6, 13.0]	56.0 [−0.3, 18.4]	[3.0, 42.0]	88.5 [3.1, 37.6]	80.6 [−3.0, 45.4]
	2-unit	[1.0, 36.0]	96.3 [0.6, 36.9]	56.9 [−13.0, 28.9]	[1.0, 87.0]	66.2 [0.9, 130.7]	59.9 [−27.9, 69.8]

Neuropsychiatry	1-unit	[1.0, 14.0]	85.6 [1.1, 12.3]	41.6 [−7.0, 24.2]	[4.0, 25.0]	90.5 [3.7, 26.9]	51.7 [−5.0, 35.6]
	2-unit	[1.0, 46.0]	85.7 [0.4, 40.1]	56.4 [−23.3, 40.1]	[1.0, 56.0]	76.3 [1.4, 72.6]	67.8 [−24.7, 55.7]

Nephrology	1-unit	[1.0, 7.0]	85.9 [0.9, 6.2]	48.9 [−2.0, 10.3]	[5.0, 38.0]	90.8 [4.7, 41.0]	64.3 [−3.0, 48.3]
	2-unit	[1.0, 21.0]	72.0 [0.4, 15.8]	52.9 [−8.1, 16.4]	[1.0, 89.0]	72.7 [1.6, 121.2]	62.2 [−28.0, 73.7]

Obstetrics and gynecology	1-unit	[1.0, 6.0]	90.3 [1.0, 5.5]	56.5 [−1.0, 7.8]	[2.0, 19.0]	92.2 [2.6, 19.8]	67.2 [−1.0, 24.3]
	2-unit	[1.0, 13.0]	94.6 [0.4, 12.9]	61.8 [−5.0, 12.1]	[1.0, 45.0]	81.2 [0.9, 55.1]	61.1 [−14.0, 37.1]

Respiratory	1-unit	[1.0, 9.0]	85.2 [1.2, 8.1]	65.1 [−1.0, 11.3]	[3.0, 27.0]	94.4 [3.4, 28.0]	76.5 [0.0, 31.4]
	2-unit	[1.0, 21.0]	95.4 [0.5, 20.6]	58.1 [−7.5, 17.6]	[1.0, 56.0]	69.7 [1.2, 79.6]	64.2 [−15.8, 47.1]

Neurology	1-unit	[1.0, 11.0]	89.7 [1.3, 10.3]	52.5 [−3.0, 16.0]	[4.0, 50.0]	97.3 [4.3, 49.1]	77.3 [−3.0, 56.5]
	2-unit	[1.0, 26.0]	88.4 [0.5, 28.8]	63.5 [−12.9, 25.7]	[1.0, 107.0]	64.3 [1.3, 165.5]	61.0 [−32.7, 86.3]

Cardiovascular medicine	1-unit	[1.0, 8.0]	85.4 [1.0, 7.0]	40.9 [−4.0, 13.1]	[4.0, 38.0]	90.6 [4.4, 41.1]	69.5 [−2.0, 46.9]
	2-unit	[1.0, 24.0]	74.2 [0.4, 18.5]	56.9 [−11.9, 21.4]	[1.0, 90.0]	71.4 [1.5, 125.1]	60.5 [−26.1, 71.3]

Hematology	1-unit	[1.0, 7.0]	94.8 [1.3, 7.0]	53.9 [−1.0, 10.1]	[5.0, 50.0]	92.9 [4.3, 52.7]	59.6 [−8.0, 67.5]
	2-unit	[1.0, 16.0]	95.1 [0.6, 16.3]	64.6 [−7.0, 15.8]	[1.0, 141.0]	76.1 [1.2, 184.7]	55.8 [−45.9, 105.3]

Gastroenterology	1-unit	[1.0, 6.0]	98.0 [1.1, 6.0]	48.0 [−1.0, 9.4]	[4.0, 35.0]	95.5 [4.2, 36.3]	81.9 [1.0, 38.8]
	2-unit	[1.0, 18.0]	76.7 [0.4, 14.5]	55.6 [−6.9, 14.9]	[1.0, 70.0]	64.9 [1.4, 106.7]	64.4 [−18.3, 57.9]

Diabetes medicine	1-unit	[1.0, 9.0]	88.8 [1.1, 8.2]	53.2 [−2.0, 13.1]	[6.0, 54.0]	94.0 [6.1, 57.0]	71.6 [−2.0, 65.0]
	2-unit	[1.0, 26.0]	83.7 [0.4, 22.4]	54.7 [−10.1, 20.8]	[1.0, 130.0]	74.1 [2.0, 173.7]	58.9 [−35.7, 98.5]

Otolaryngology	1-unit	[1.0, 8.0]	93.1 [1.4, 8.1]	58.0 [−1.0, 11.1]	[4.0, 38.0]	89.9 [4.3, 41.4]	68.8 [−2.0, 47.4]
	2-unit	[1.0, 18.0]	90.0 [0.6, 19.5]	64.2 [−7.1, 17.1]	[1.0, 84.0]	64.9 [1.4, 128.4]	64.3 [−26.8, 72.2]

Orthopedic surgery	1-unit	[1.0, 7.0]	90.4 [1.1, 6.5]	69.3 [0.0, 8.7]	[5.0, 40.0]	93.2 [4.8, 42.3]	64.2 [−3.0, 51.6]
	2-unit	[1.0, 16.0]	95.6 [0.5, 15.9]	58.1 [−5.0, 13.2]	[1.0, 102.0]	80.3 [1.6, 126.1]	58.8 [−30.6, 78.9]

Ophthalmology	1-unit	[1.0, 3.0]	87.5 [0.8, 3.1]	22.4 [−2.0, 6.9]	[3.0, 37.0]	95.5 [3.5, 38.1]	55.2 [−10.0, 51.6]
	2-unit	[1.0, 8.0]	66.6 [0.4, 6.0]	37.2 [−7.2, 11.6]	[1.0, 75.0]	59.0 [1.0, 126.3]	60.3 [−40.4, 82.3]

Dermatology	1-unit	[1.0, 7.0]	82.7 [1.1, 6.0]	63.1 [−1.0, 8.5]	[3.0, 29.0]	97.8 [3.3, 29.3]	62.2 [−4.0, 37.8]
	2-unit	[1.0, 15.0]	91.8 [0.4, 14.4]	59.4 [−5.5, 13.2]	[1.0, 80.0]	90.8 [1.1, 87.9]	55.1 [−24.5, 58.6]

Cardiovascular surgery	1-unit	[1.0, 7.0]	99.1 [1.0, 7.0]	35.1 [−4.0, 13.1]	[4.0, 40.4]	91.2 [4.0, 43.5]	54.5 [−8.0, 58.1]
	2-unit	[1.0, 26.0]	68.4 [0.4, 18.5]	54.0 [−11.8, 21.4]	[1.0, 114.0]	79.3 [1.2, 143.2]	58.0 [−41.8, 91.4]

Gastrointestinal Surgery A	1-unit	[1.0, 7.0]	94.8 [1.2, 6.9]	55.0 [−1.0, 9.9]	[3.0, 29.0]	95.5 [3.3, 29.9]	65.5 [−3.0, 36.7]
	2-unit	[1.0, 17.0]	93.9 [0.5, 16.5]	60.9 [−6.7, 15.4]	[1.0, 67.0]	74.6 [1.1, 89.3]	62.0 [−22.3, 56.4]

Gastrointestinal Surgery B	1-unit	[1.0, 8.0]	91.2 [1.2, 7.6]	50.0 [−2.0, 12.0]	[3.0, 30.0]	90.7 [3.4, 32.3]	71.3 [−1.0, 36.9]
	2-unit	[1.0, 21.0]	87.6 [0.5, 19.0]	59.8 [−9.3, 19.1]	[1.0, 70.0]	70.2 [1.1, 99.1]	60.9 [−20.1, 55.9]

Urology	1-unit	[1.0, 6.0]	88.9 [1.0, 5.5]	52.2 [−1.0, 8.6]	[3.0, 36.0]	95.7 [3.6, 36.8]	69.9 [−3.0, 44.2]
	2-unit	[1.0, 15.0]	80.2 [0.4, 12.7]	58.8 [−6.4, 13.6]	[1.0, 81.0]	68.9 [1.1, 116.9]	61.9 [−27.3, 68.1]

Neurosurgery	1-unit	[1.0, 9.0]	94.5 [1.3, 8.8]	53.4 [−2.0, 13.0]	[4.0, 37.0]	93.5 [4.4, 38.9]	66.7 [−3.0, 46.5]
	2-unit	[1.0, 23.0]	96.4 [0.5, 23.3]	59.9 [−9.4, 20.4]	[1.0, 87.0]	74.3 [1.5, 116.1]	61.3 [−27.3, 71.1]

Emergency A	1-unit	[1.0, 15.0]	92.3 [1.7, 14.6]	57.9 [−3.0, 21.2]	—	—	—
	2-unit	[1.0, 43.0]	98.5 [0.6, 42.8]	55.4 [−15.4, 33.4]	—	—	—

Emergency B	1-unit	[1.0, 13.0]	93.6 [1.5, 13.3]	47.8 [−4.0, 21.1]	—	—	—
	2-unit	[1.0, 40.0]	97.2 [0.5, 39.4]	57.5 [−17.2, 33.9]	—	—	—

Pediatrics	1-unit	[1.0, 8.0]	88.3 [1.4, 7.6]	62.5 [−1.0, 10.2]	—	—	—
	2-unit	[1.0, 16.0]	88.3 [0.6, 17.6]	66.1 [−6.1, 15.6]	—	—	—

Pediatrics surgery	1-unit	[1.0, 7.0]	92.9 [1.2, 6.8]	68.0 [0.0, 8.8]	—	—	—
	2-unit	[1.0, 16.0]	96.8 [0.5, 16.0]	59.4 [−4.9, 13.4]	—	—	—

*Note:* For the sensor alert dataset, for wards with < 100 data points, calculations of the correct similarity could not be performed, and they were therefore excluded from consideration. Numbers in bold indicate greater similarity. *n*: number of data. *m*^×^/*s*: the times–divide expression. *μ* ± *σ*: the plus–minus expressions.

## Data Availability

The data used in this study are not publicly available. However, nurse call logs are available at each facility.

## Supporting Information

The data for the ward that could not be included in Figure 5 are presented in Supporting Figure 1.

In addition, Supporting Figure 2 shows the results of evaluating the relative errors of statistical estimates based on extremely small random samples (0.01% and 0.1%) of nurse call data.

These results suggest that, under severely limited sample sizes, the times–divide expression may yield more reliable estimates than the plus–minus expression.
