# Supplementary material for: The Times–Divide Expression: An Intuitive Approach for Describing Right-Skewed Data in Nursing Practice
Source: J Nurs Manag. 2025 Nov 14;2025:3434734. doi: 10.1155/jonm/3434734 (PMC12638163; doi:10.1155/jonm/3434734)
Supplement: Supporting Information — Additional supporting information can be found online in the Supporting Information section. Supporting Information. [file 3434734.f1.zip › Supporting Figure 1_Ward Data Excluded from Figure 5.pdf]

All figures except for Fig. 5 are shown below.

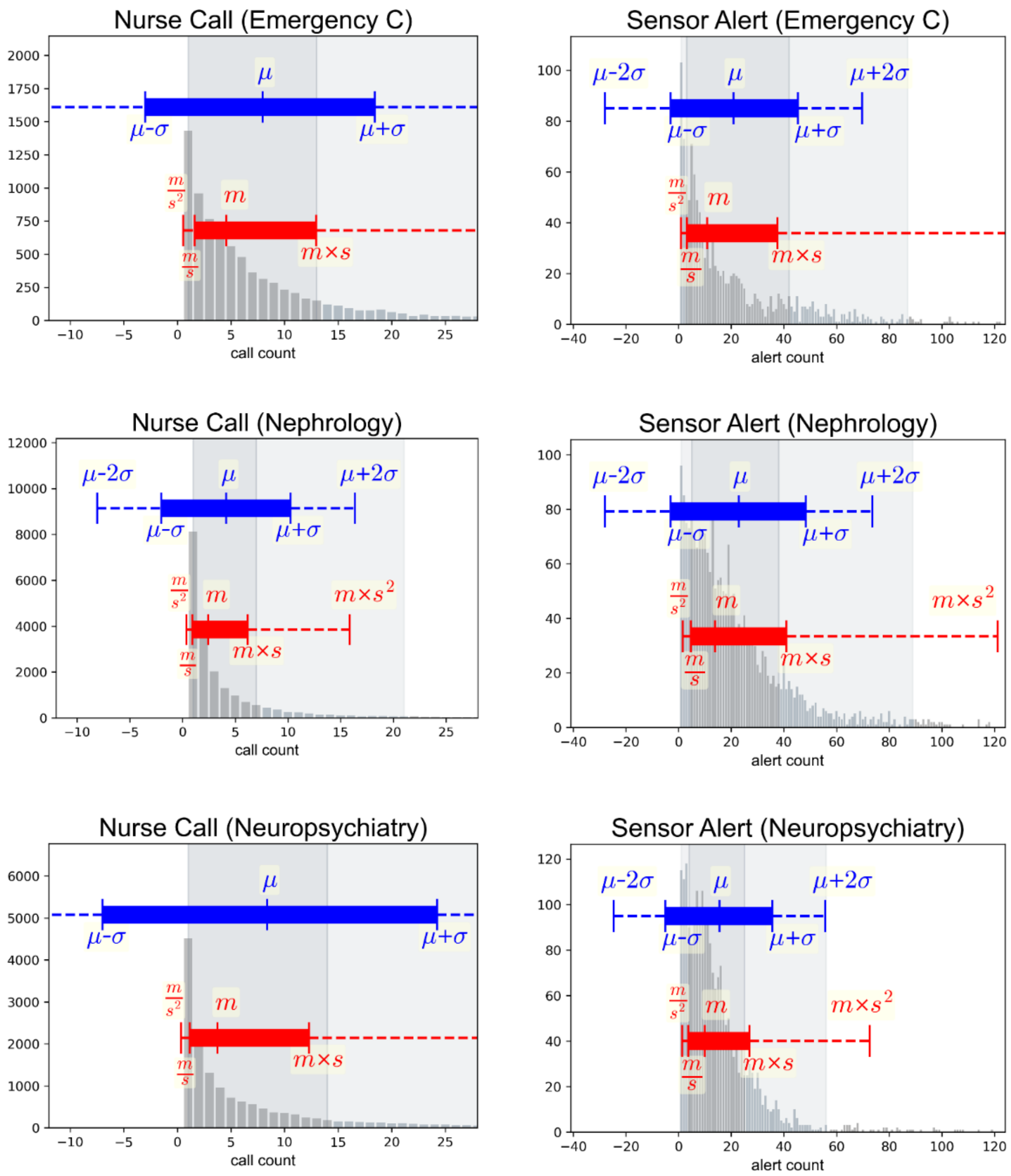

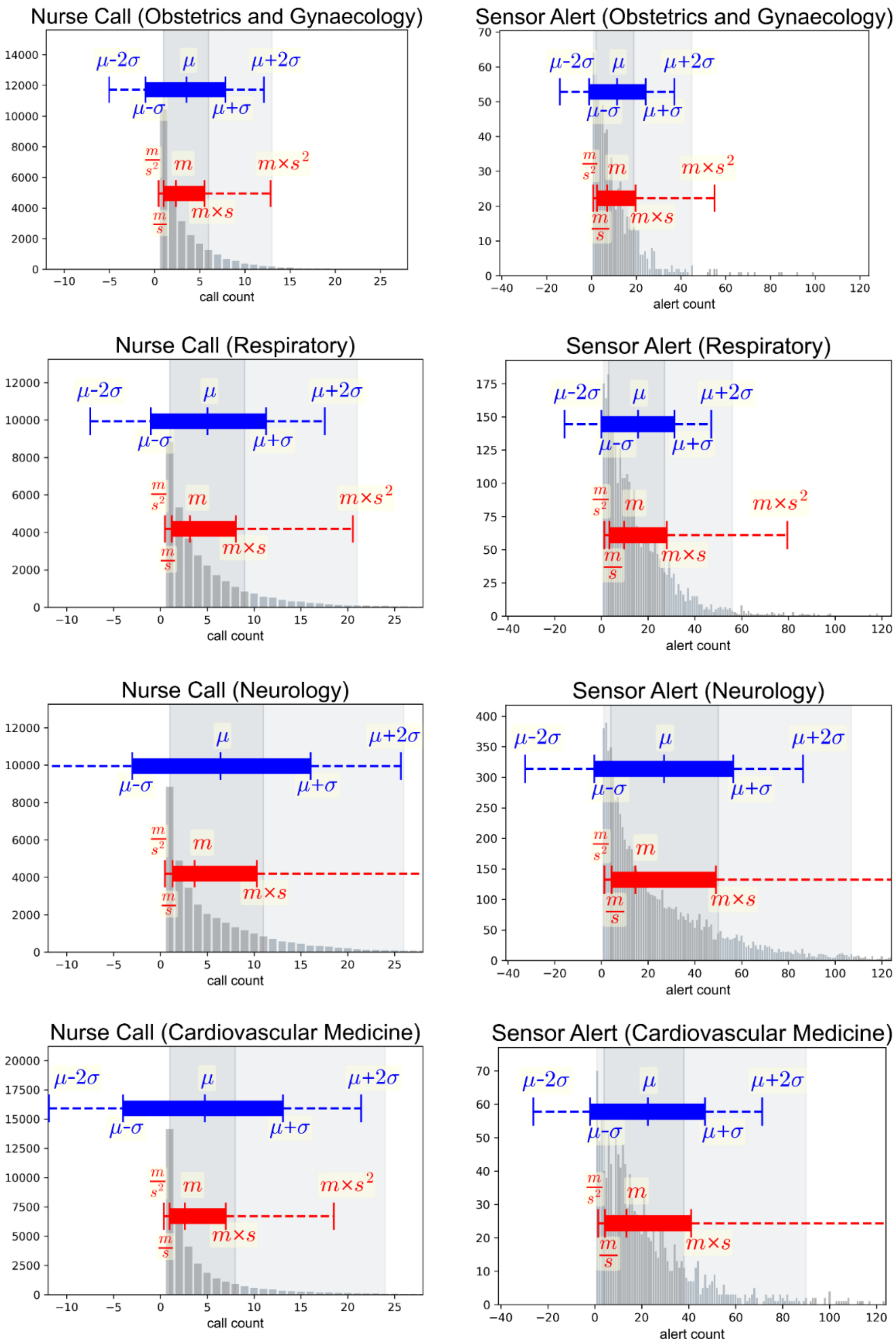

Nurse Call (Obstetrics and Gynaecology)
call count
Sensor Alert (Obstetrics and Gynaecology)
alert count
Nurse Call (Respiratory)
call count
Sensor Alert (Respiratory)
alert count
Nurse Call (Neurology)
call count
Sensor Alert (Neurology)
alert count
Nurse Call (Cardiovascular Medicine)
call count
Sensor Alert (Cardiovascular Medicine)
alert count
μ-2σ
μ
μ+2σ
μ-σ
μ+σ
$\frac{m}{s^2}$
m
$m \times s^2$
$\frac{m}{s}$
$m \times s$

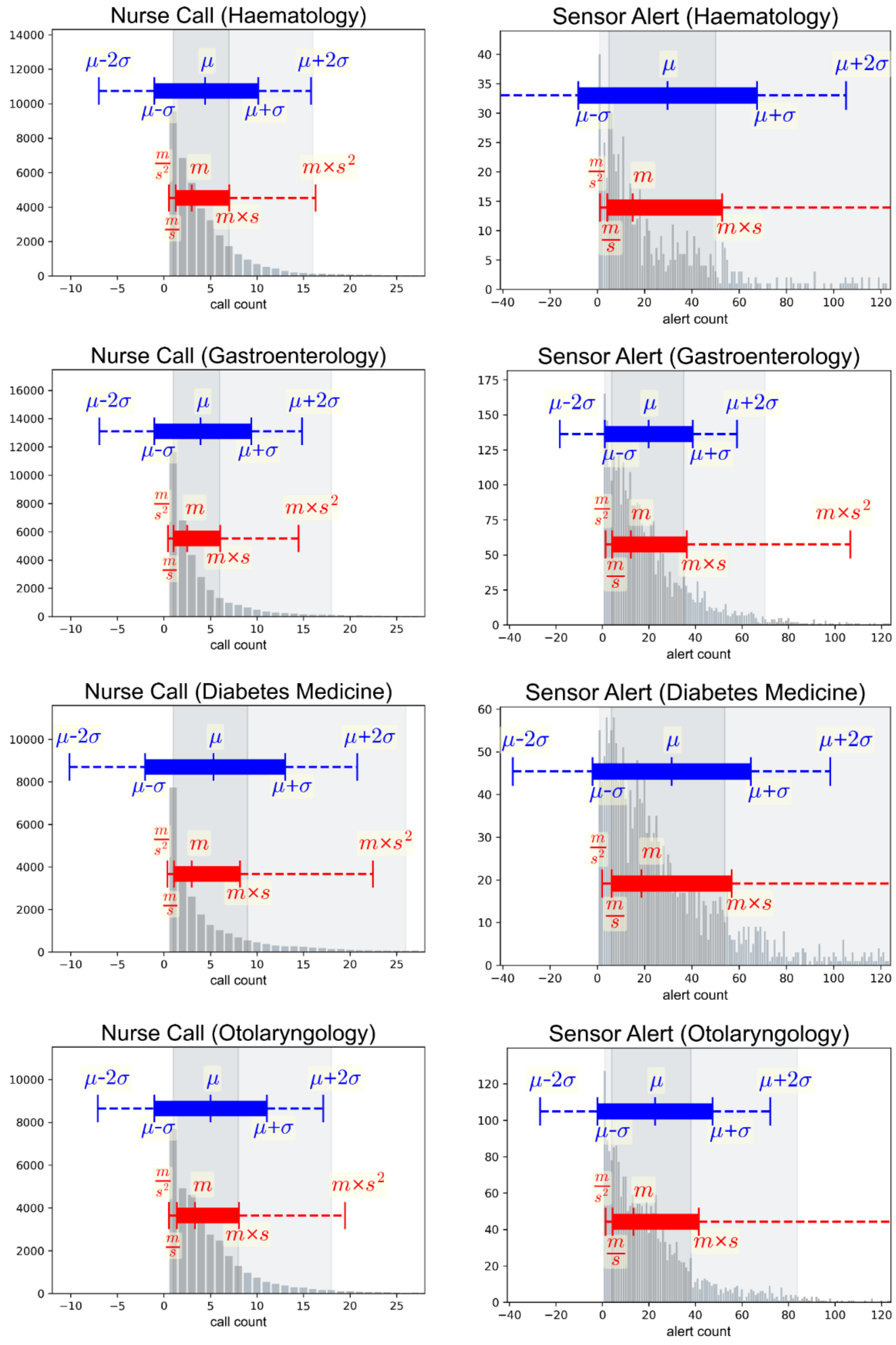
Nurse Call (Haematology)
call count
Sensor Alert (Haematology)
alert count
Nurse Call (Gastroenterology)
call count
Sensor Alert (Gastroenterology)
alert count
Nurse Call (Diabetes Medicine)
call count
Sensor Alert (Diabetes Medicine)
alert count
Nurse Call (Otolaryngology)
call count
Sensor Alert (Otolaryngology)
alert count
μ-2σ
μ-σ
μ
μ+σ
μ+2σ
$\frac{m}{s^2}$
$\frac{m}{s}$
m
$m \times s$
$m \times s^2$

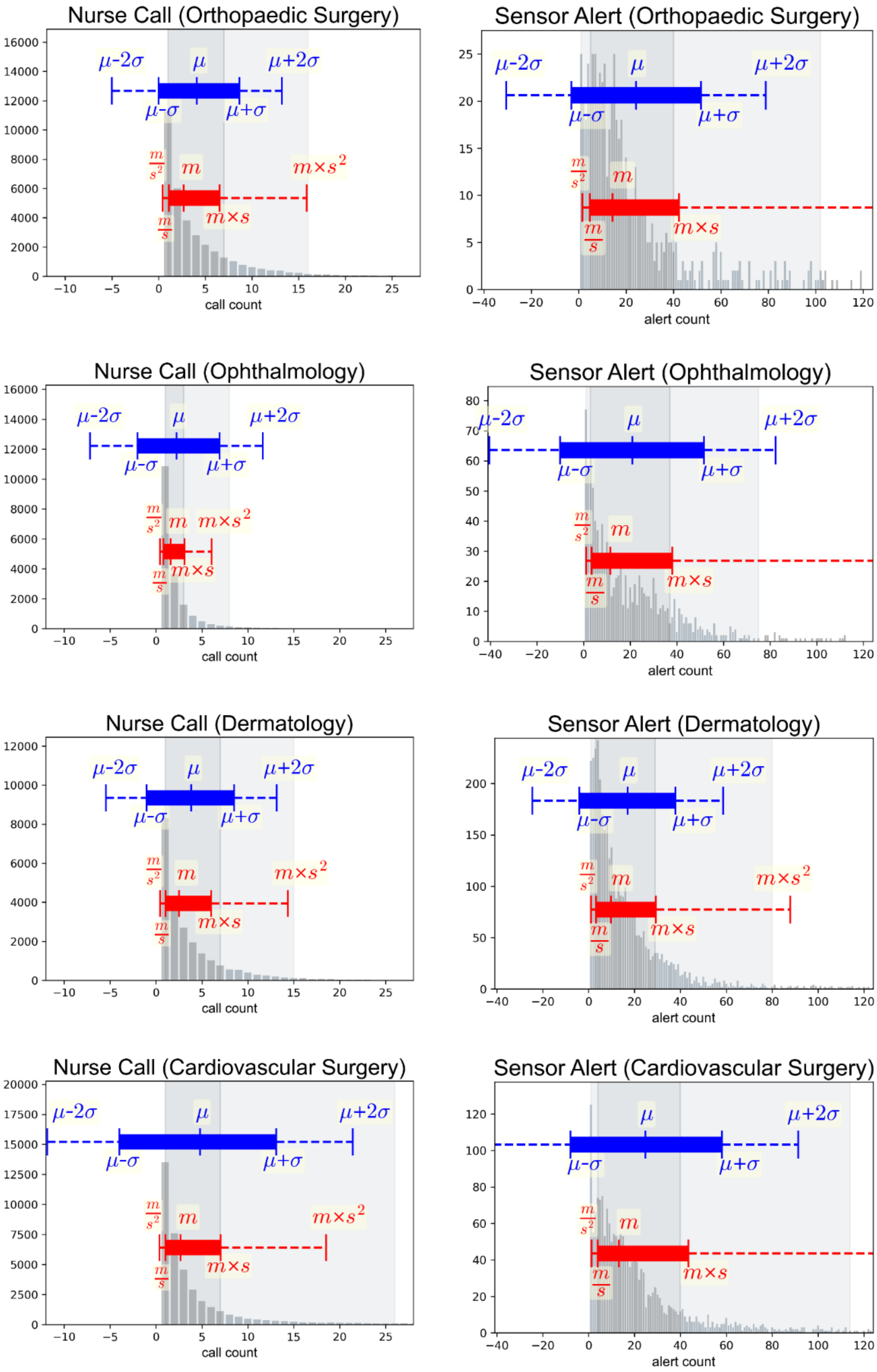
Nurse Call (Orthopaedic Surgery)
call count
Sensor Alert (Orthopaedic Surgery)
alert count
Nurse Call (Ophthalmology)
call count
Sensor Alert (Ophthalmology)
alert count
Nurse Call (Dermatology)
call count
Sensor Alert (Dermatology)
alert count
Nurse Call (Cardiovascular Surgery)
call count
Sensor Alert (Cardiovascular Surgery)
alert count
μ-2σ
μ-σ
μ
μ+σ
μ+2σ
m/s²
m/s
m
m×s
m×s²

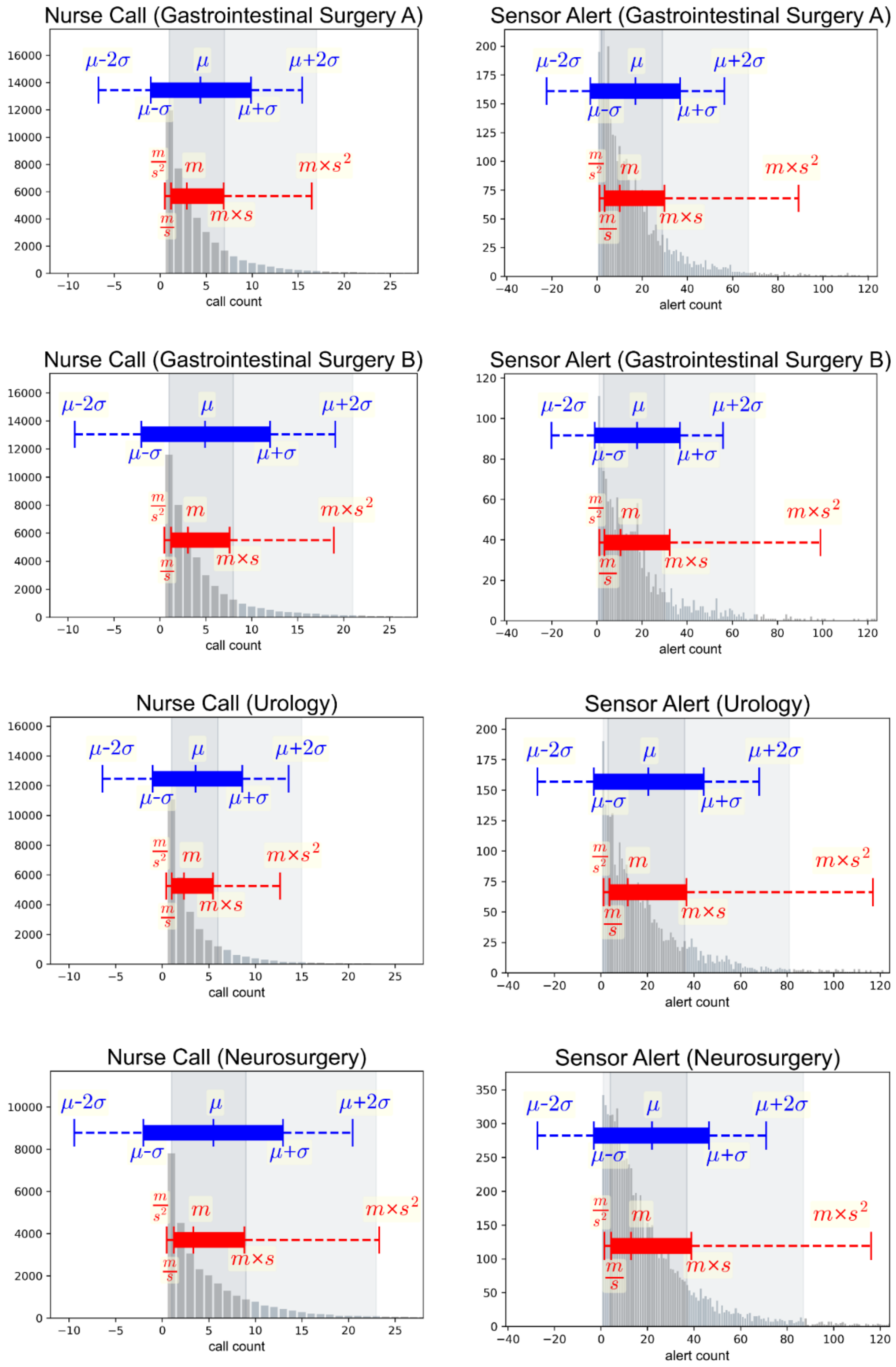
Nurse Call (Gastrointestinal Surgery A)
Sensor Alert (Gastrointestinal Surgery A)
Nurse Call (Gastrointestinal Surgery B)
Sensor Alert (Gastrointestinal Surgery B)
Nurse Call (Urology)
Sensor Alert (Urology)
Nurse Call (Neurosurgery)
Sensor Alert (Neurosurgery)
μ-2σ
μ
μ+2σ
μ-σ
μ+σ
m/s²
m
m×s²
m/s
m×s
call count
alert count

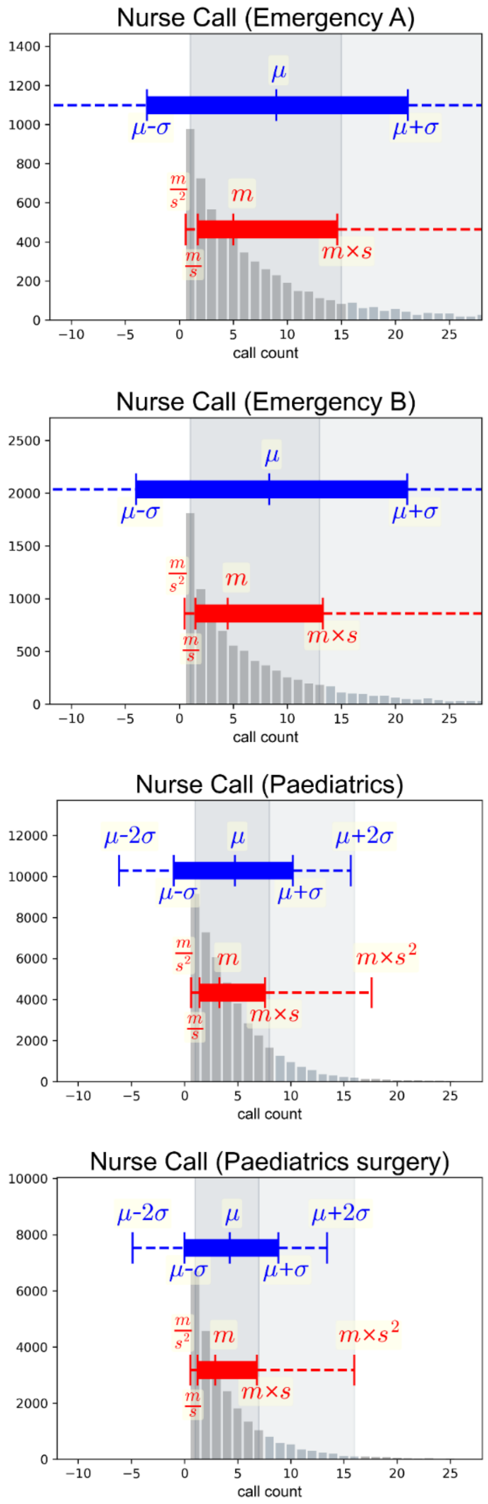
Nurse Call (Emergency A)
Nurse Call (Emergency B)
Nurse Call (Paediatrics)
Nurse Call (Paediatrics surgery)
call count
μ
μ-σ
μ+σ
μ-2σ
μ+2σ
m
m/s
m/s²
m×s
m×s²
