# Supplementary material for: The Times–Divide Expression: An Intuitive Approach for Describing Right-Skewed Data in Nursing Practice
Source: J Nurs Manag. 2025 Nov 14;2025:3434734. doi: 10.1155/jonm/3434734 (PMC12638163; doi:10.1155/jonm/3434734)

This figure shows the results of evaluating the relative errors of statistical estimates derived from extremely small random samples (0.1% and 0.01%) of nurse call data. For each sampling ratio, 1000 iterations were performed, and in each iteration, the relative error was calculated as the absolute difference between the sample statistic and the population statistic, divided by the absolute value of the population statistic.

The two types of statistical expressions compared were the plus-minus expression and the times-divide expression.

Across all evaluated statistics, the times-divide expression consistently produced smaller and more stable relative errors than the plus-minus expression. These results suggest that the times-divide expression may provide more reliable estimates under extremely limited sample sizes.

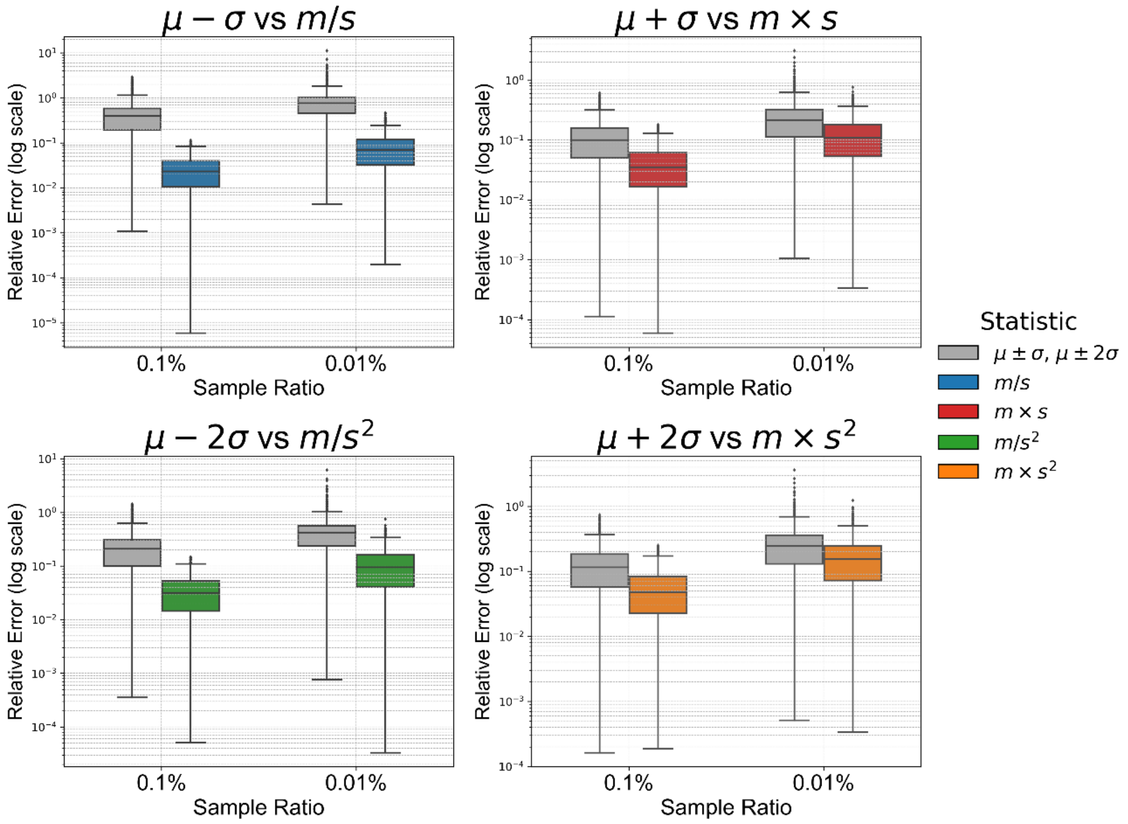

Supplement: Supporting Information — Additional supporting information can be found online in the Supporting Information section. Supporting Information. [file 3434734.f1.zip › Supporting Figure 2_Relative Errors of Statistical Estimates from Extremely Small Random Samples.pdf]
